# Extending Pharmacist Roles in Primary Healthcare to Meet the Needs of Universal Health Coverage in Zimbabwe: A Pharmacist Perspective and Curriculum Evaluation

**DOI:** 10.3390/pharmacy10030054

**Published:** 2022-05-13

**Authors:** Douglas Chiutsi, Fatima Suleman, Velisha Ann Perumal-Pillay

**Affiliations:** Discipline of Pharmaceutical Sciences, College of Health Sciences, University of KwaZulu-Natal, Private Bag X54001, Durban 4000, South Africa; dougiechiutsi@gmail.com (D.C.); sulemanf@ukzn.ac.za (F.S.)

**Keywords:** pharmacist perspectives, extending pharmacist roles, primary healthcare, pharmacy curriculum, universal health coverage, Zimbabwe

## Abstract

Zimbabwean pharmacists undergo university level education to understand the biochemical mechanisms and actions of medicines but are limited in their scope of practice. They are called medicines experts, yet they are not allowed to apply their specialized knowledge independently in direct patient management. We aim to obtain Zimbabwean pharmacists’ perceptions on extending their scope of practice and to evaluate the Zimbabwe pharmacy honours degree curriculum to determine the competencies covered and whether these are in-line with an extended scope of practice. Qualitative semi-structured interviews with selected pharmacists were conducted to gather perspectives on the BPharm (Hons) curricula and extending pharmacists’ scope of practice. A desktop review of the pharmacy curricula was also conducted to determine competencies covered. The results showed that pharmacists are keen to extend their scope of practice but the curriculum does not equip them with the required exit level competencies. “*The pharmacist is obviously not equipped currently but needs to be involved in direct patient care such as identifying and managing medicine therapy problems, prescription extension, ordering and reviewing laboratory data and administration* *of vaccines and immunizations*”. There exists an opportunity for pharmacists to extend their scope of practice in order to achieve universal health coverage.

## 1. Introduction

The number of licenced pharmacists and pharmacies in Zimbabwe has almost tripled in the past 10 years [1,2]. The number of pharmacies across the country has increased from 287 in 2011 to 933 in 2020 [1,2]. The number of licenced pharmacists has also increased from 567 (0.5/10,000) in 2011 to 1251(0.9/10,000) in 2020 [1,2].

Pharmacists are health professionals who focus on safe and effective medication use [3]. Pharmacists undergo university level education to understand the biochemical mechanisms and actions of medicines, medicines use, therapeutic roles, side effects, potential medicine interactions, and monitoring parameters, and also interpret and communicate this specialised knowledge to patients, physicians, and other healthcare providers [3]. The main duties of a pharmacist in Zimbabwe are processing of prescriptions; dispensing of medications to patients; providing instruction and information regarding correct use of medications supplied; adverse medicine reactions and administration requirements of patients’ medications; data collection for reporting and ordering stock purposes; and stock management and control [1,4]. Zimbabwean pharmacists only dispense medications according to the doctor’s prescription and have no authority to diagnose and prescribe independently, renew/extend prescriptions, change drug dosage/formulation, make therapeutic substitution, prescribe for minor ailments, initiate prescription drug therapy, order and interpret laboratory tests, and or administer a medicine by injection [3,4]. As countries move towards Universal Health Coverage (UHC), equitable access to medicines will become a key issue. Pharmacists should therefore move from behind the counter and start serving the public by providing pharmaceutical care instead of medicines only [5]. There exists a limited future in terms of just dispensing, as that activity can potentially be taken over by dispensing machines, and/or technicians [5]. Pharmacists have been trained to do more than they currently are licensed to do, and this enables them to better serve the community than they currently do [5] under a universal health coverage model.

Zimbabwe joined the Universal Health Coverage partnership (UHC-P) in 2018, which supports capacity building for human resources for health and strengthening information systems [6]. The World Health Organisation (WHO) defines Universal Health Coverage (UHC) as the provision of promotive, preventive, curative, rehabilitative, and palliative health services to all people and communities [6,7]. The services should be of sufficient quality to be effective, while also ensuring that the use of these services does not expose the user to financial hardship [6]. As part of expanding access, the traditional role of the pharmacist is rapidly evolving globally [8,9,10,11]. Pharmacists are developing skills and expertise in evidence-based practice and pharmaceutical care, which are enabling them to assume new roles with a focus on patient care [9,11,12,13,14]. Extended pharmacist services are referred to those services that are not associated with traditional services offered by the pharmacists such as dispensing and providing individual consultations on prescription and/or over the counter (OTC) medications; but include a new series of services in the management of the patient [8]. These services may include for example medication therapy management; home medication review, which involves comprehensive medication reviews to look for medication-related problems; and all aspects of chronic disease management. Extended pharmacist services may also include non-communicable disease screening, patient education and knowledge, disease monitoring, and communication with the primary healthcare team [8].

Due to the increasing number of pharmacists in Zimbabwe, universal health coverage can be a step closer if Zimbabwe can tap into the knowledge of pharmacists and apply it directly to patients to improve patient health access and outcomes. The pharmacist is the most accessible healthcare practitioner at a lower cost [8,15] as compared to other healthcare practitioners, and by extending pharmacist services, universal health coverage can be achieved sooner. Some of the extended pharmacist services that can be absorbed are prescription authority; injection authority; ordering and interpreting a limited set of laboratory tests; and adapting and or managing a prescription [11]. Injection authority will allow pharmacists to take an active role in routine immunisations and vaccinations, and in light of the COVID-19 pandemic, pharmacist’ initiated vaccinations will greatly improve access. Prescription authority can be used in the treatment of minor ailments/conditions, for smoking/tobacco cessation programmes, and in emergencies [8,12]. Pharmacists should be able to independently or in a collaborative practice make therapeutic substitution; change drug dosage, formulation or regimen; and renew/extend a prescription for continuity of care [8]. Pharmacists should also be able to order and interpret limited laboratory tests so that they can make informed recommendations and decisions based on available evidence [9,14,15].

Extending pharmacist services will help in providing for a health system with checks and balances at all levels of care for it will confer responsibility for patient healthcare outcomes to all the practitioners managing a patient and may also improve communication between healthcare professionals [8,9]. A study conducted by Guillaume et al. (2004) reported that resistance by other healthcare professionals to extending pharmacists services has less to do with competencies of the pharmacists but more to do with the discomfort of encroachment of professional turfs and boundaries [12].

Other countries such as the United States of America and Canada have adopted regulatory changes that permit pharmacists to provide a much greater range of services [11,12]. By embracing these services, the governments are sending a clear signal that pharmacists can offer accessible, quality care in a number of service areas, often at less cost than the typical physician or hospital setting [13]. Pharmacist-extended services would help improve patient access to their medications at a lower cost, optimization of medication management, better resource utilization, better utilization of pharmacist skills, and ease the burden on general practitioners [13].

Extended pharmacist services may require additional training or special skills, knowledge, and/or facilities [8,9,13,14], thus the aim of this research was to investigate Zimbabwean pharmacists’ opinions on extending their scope of practice and to evaluate the two Zimbabwe Pharmacy Honours Degree curricula to determine the competencies covered and whether these are in line with an extended scope of practice.

## 2. Materials and Methods

**Figure 1:**
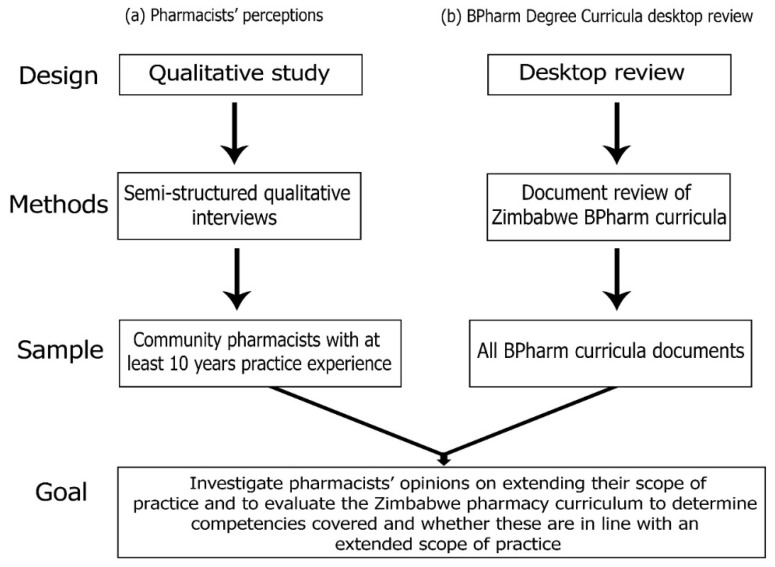
Outline of the study.

The study employed a triangulation process by employing a mixed method study design. The first arm of the study (a) involved qualitative semi-structured interviews and the second arm (b) involved a comprehensive document review of the two Zimbabwe BPharm degree curricula as outlined in Figure 1 below.

### 2.1. Arm a: Pharmacists’ Perceptions

#### 2.1.1. Study Design and Setting

This first part of the study was conducted as qualitative semi-structured interviews with community pharmacists in Zimbabwe. The interviews were conducted from October to December 2020.

#### 2.1.2. Study Population and Sampling Strategy

A list of all registered community pharmacists was obtained from the Pharmacists Council of Zimbabwe. A list of registered Pharmacists for 2020 and a list of pharmacists who were on the pharmacist main register in 2010 were obtained from the Pharmacists Council of Zimbabwe to make sure that participants had more than 10 years of practical experience. The number of pharmacists in 2020 who were on the main register of pharmacists in 2010 was 410. A total of 33 pharmacists were shortlisted for interviews using purposive methods by targeting and identifying those individuals who had experience in community pharmacies and were influential about service development. A snowball technique was further employed in that the identified individuals could suggest another pharmacist (who met inclusion criteria) to participate in the study, if they chose not to.

#### 2.1.3. Development of the Data Collection Tool

A semi-structured interview guide with questions and probes was used to stimulate expert views and opinions on the pharmacy degree programme and pharmacist’s opinions on changing their roles to meet the needs of Universal Health Coverage. The interviews were carried out by telephone and not face to face, due to COVID-19 restrictions. The interview guides probed pharmacists on:

(i) Perceived knowledge on universal health coverage and the current role of the Zimbabwean pharmacist
(ii) Pharmacist views on extending Zimbabwean pharmacists’ roles in patient care
(iii) Pharmacists’ views on the BPharm curricula and if it equipped pharmacists with exit level competencies supporting extended pharmacist roles in patient care
(iv) Pharmacists’ desired roles in the Zimbabwe healthcare system to achieve universal health coverage.

The interview guide was piloted on three community pharmacists to establish face validity and was modified accordingly. These pilot pharmacists were not included in the purposeful sampling process and therefore were not included in the analysis.

#### 2.1.4. Procedure for Carrying out Interviews

The pharmacists were contacted by telephone and email or WhatsApp with a copy of request and consent to participate in the research project and the interview guide questions. A follow up phone call was done to obtain consent or suggestion for an alternate person to meaningfully contribute to the study. A time was then arranged for the interview. The interview questions and the informed consent form were sent via email and or WhatsApp to the informant at least 2 days before the scheduled date. Before the day of the interview, the informant emailed back the signed informed consent to participate in the study. All participants who agreed to be interviewed gave their oral and written, informed consent before participating in the study.

#### 2.1.5. Data Collection and Analysis

The interviews were conducted in English (pharmacists are generally good at speaking English, as the medium of instruction in institutions and universities in Zimbabwe is exclusively in the English language). Each interview lasted for 18 to 25 min. Probing questions were asked, and participants were given freedom to express additional views and comments. The interviews were audio-recorded and coded. The recordings were downloaded to a password-protected computer and the phone audio record cleared. The interviews were then transcribed verbatim. Transcripts were clarified via email or phone with participants to determine if information was accurately captured. The transcripts were managed using NVivo12 and were coded and analysed using interpretive thematic analysis [16]. The themes were then finalised by collapsing categories under descriptive titles that reflected the content of the data and the aim of the study. Illustrative quotes, which represented pharmacists’ opinions and experiences, were selected for reporting and appear in the text in italics.

### 2.2. Arm b: Curriculum Review

A review of the Zimbabwe pharmacy curricula leading to registered pharmacists identifying the overall differences with a focus on the amount of time devoted for patient care, public health, and pharmacy practice aspects, was conducted. The syllabi of the BPharm curricula were obtained from the Pharmacists Council of Zimbabwe. This was a desktop review conducted from October to December 2020. Zimbabwe had only two pharmacy schools training pharmacists, hence two curricula were applicable to the study. To maintain anonymity, the two pharmacy programmes were coded BPharm (Hons) 1 and BPharm (Hons) 2.

To achieve the objective of the study, the curriculum content from each programme were divided into four core areas named as:

Area 1: Basic/Biomedical Sciences;

Area 2: Pharmaceutical Sciences;

Area 3: Social/Behavioural/Administrative Sciences;

Area 4: Clinical Sciences, as outlined in the American Appendix-B of Accreditation Council of Pharmacy Education’s (ACPE) accreditation 2016 standards and guidelines for the professional programme in pharmacy leading to the Doctor of Pharmacy degree [17]. ACPE guidelines were selected because American pharmacists have PharmD as a first professional degree and are practising with a focus on patient care [18]. The PharmD programme has significant experiential or clinical education components in introductory and advanced levels for the safe and effective use of medicines. The experiential education prepares graduates to be practice ready, as they already have spent a significant amount of time training in areas of direct patient care and research. The PharmD programme supports an extended pharmacist role towards achieving UHC, although its length and intensity of study have notable differences from the BPharm degree.

#### 2.2.1. Data Collection and Analysis

The syllabi were analysed from each curriculum outline and compared with special focus on clinical sciences and social/behavioural/administrative sciences areas. The number of hours of each core area was separated as described in ACPE’s accreditation standards and guidelines and the American Pharmacy Curriculum Outcomes Assessment guidelines [17,19,20]. Subjects from each programme were divided into theory lectures and practical hours. The duration of time spent (Total Hours) on each subject was used to determine pharmacist key competencies.

#### 2.2.2. Conversion of Credits into Hours

The first curriculum (BPharm (Hons) 1) is defined in number of hours. The second curriculum (BPharm (Hons) 2) is defined in credits, where each credit is equal to 2 h, hence both curricula were converted into number of hours to enable ease of comparison.

#### 2.2.3. Division of Core Curriculum Content and Comparison

The core areas from each programme were tabulated and the results were analysed and compared with a focus on area 3 and area 4, as these two directly related to patient care.

*In core area 3*: health and pharmaceutical policies, practice management, pharmaco-economics, pharmaco-epidemiology, pharmacy law, regulatory affairs, ethics, professional communication, and social and behavioural aspects of practice both in community and hospital pharmacy practice were collated. This area is very important for public health and its programmes.

*In core area 4*: clinical pharmacy, pharmacotherapy, therapeutics, drug information, medication safety, and literature evaluation aspects were collated. Special focus was made to see if there was emphasis on patient care, for example, efforts to improve therapeutic outcomes by counselling, disease and medicine management, and maintaining patient profiles.

### 2.3. Ethical Approval

The study was approved by the University of KwaZulu-Natal (UKZN) Biomedical Research Ethics Committee (BE465/19) and gatekeeper approval was obtained from the Pharmacists Council of Zimbabwe.

## 3. Results

### 3.1. Pharmacists’ Perceptions

A total of 10 pharmacists were interviewed (Table 1). Though 33 Pharmacists were shortlisted for interviews, 8 pharmacists were unreachable and therefore could not be contacted, 9 pharmacists suggested other pharmacists who could contribute more meaningfully to the project, 7 pharmacists rejected to participate in the project, and 10 Pharmacists agreed to participate. Data saturation was reached after six interviews. Among the 10 pharmacists interviewed, 8 were male and 2 were female. Seven pharmacists were pharmacy owners and three were employee managers. The average interview lasted approximately 22 min. From thematic analysis of data, six major themes (with subthemes) emerged. These are discussed below.

#### 3.1.1. Theme 1: Understanding UHC

Pharmacists were able to define the term universal health coverage and exhibited an understanding of the concept and its importance to the population at large as noted in the verbatim quotes below:

“*Universal health coverage is the concept of ensuring that everybody is able to access good quality health in a way that is affordable, and which is acceptable*”.

“*Ensuring that everybody is able to access health without financial hardship or strain*”.

#### 3.1.2. Theme 2: Current Pharmacist Role in the Community

Pharmacists highlighted that their main role currently is the dispensing of medication and counselling of patients on prescribed medicines. Point of care diagnostic testing such as Blood Pressure screening, Glucose testing, and Malaria and HIV tests are also carried out. Pharmacists described their roles as follows:

“*The main duties of a retail pharmacist is dispensing medicines to patients. We also counsel patients on prescribed medication and break bulk to meet specific customer needs*”.

“*I would say dispensing accounts for 95% of our duties, the other 5% is patient counselling, point of care diagnostics tests and stock management*”.

#### 3.1.3. Theme 3: Potential Extended Roles of Community Pharmacists

##### Providing Vaccines and Immunisations

Pharmacists demonstrated an understanding of how medicines were manufactured, stored, and the supply chain to follow before use by the patient. They are keen on providing vaccines and immunisations to the population after extra training as noted in the verbatim quotes below:

“*With the current curriculum pharmacists are obviously not equipped to administer injections but with appropriate training, they can effectively administer immunisations and vaccines just like village health workers some without even O Levels* (“O Level” examinations are taken after four years of secondary school) *but can test for malaria and prescribe Coartem [artemether/lumefantrine tablets] in the rural areas after undergoing training*”.

“*A three-day course is enough to recap and educate pharmacists on how vaccines and immunisations can be administered*”.

##### Pharmacist Physical Examinations and Prescribing in Minor Ailments, HIV/AIDS

Participants’ views on pharmacist physical examinations were mixed. One pharmacist highlighted that examinations would need skills beyond those of a pharmacist as some of these examinations are quite invasive. Physical examinations may also require other tests to confirm the diagnosis, for example checking for lumps in breast examinations is verified by other means as benign or malignant. Their perceptions are captured in the verbatim quotes below:

“*Pharmacists have been doing minor physical examinations including ear and eye check, tonsillitis, minor injuries and burns and recommending over the counter treatments and pharmacist-initiated medicines. Where there is need for further examinations, they have always referred to the appropriate cadre for further assistance*”.

“*Minor ailments must be fully described and the management options fully described and documented into an Essential Drug List for Pharmacists with clear standard treatment guidelines and protocols for referral*”.

##### Ordering and Reviewing Laboratory Data for Monitoring Services in the Management of Disease Conditions

Pharmacists indicated that they have the knowledge and skill to request patient laboratory tests and to interpret laboratory data in the monitoring and management of chronic diseases. Pharmacists also acknowledged the need for continuous education and more practical experience.

“*I understand that pharmacists are using rapid diagnostic tests in the pharmacy to check for BP (blood pressure), cholesterol, HIV, malaria amongst other tests. These point of care diagnostics may not be the gold standard but pharmacists are already ordering these tests in their premises and interpreting the results*”.

“*Pharmacists should be involved directly with patient care and allowed to take responsibility for their actions in improving patient health outcomes. Pharmacists should be allowed to order glycated haemoglobin concentration, INR in patients on warfarin and so on. This service will definitely improve patient health outcomes and lower costs*”.

“*I think pharmacists have the capacity to order laboratory tests and review prescriptions. I would strongly recommend that pharmacists get practical courses and training in the principles behind interpretation of laboratory tests and adjustment of doses*”.

##### Change Drug Dosage/Formulation and Renew or Extend Prescriptions

The pharmacists highlighted that assuming this extended role will legalise some already existing practice expansions and will result in the implementation of standardized guidelines and protocols to be followed. These will improve patient health access, out-of-pocket expenditure, healthcare outcomes, and implementation of the extended roles. They also indicated that this extended service will definitely impact positively on patient healthcare outcomes if the pharmacists are well trained and have the confidence to carry out these tasks.

“*This practice of extending prescriptions has been going on but without monitoring the patient. Patients usually come to the pharmacy asking for their repeat drugs and pharmacists usually dispense these chronic medications without asking for the prescription as long as the patient is in their database*”.

“*Allowing pharmacists to extend physician prescriptions will confer responsibility on the pharmacist and eliminate the bad practices that have been going on*”

“*Pharmacists may not be capable or competent to extend prescriptions at the moment but that is an area that they should embrace in an effort to achieve Universal Health Coverage*”.

#### 3.1.4. Theme 4: Barriers to Extended Pharmacist Services

The barriers to adoption of extended pharmacist services identified by the pharmacists include: the unavailability of suitable facilities within the pharmacy such as private consultation rooms, competences of the pharmacist to assume new roles, and reimbursement models for the extended services and the pharmacy legislation that is not supportive of the extended roles. On the issue of competence, it was suggested to have a specialist register for pharmacists who take extra training and then become accredited to provide certain extended services according to their competencies and extra training.

“*I think the expanded roles are sort of specialist branches of pharmacy and would need specialisation through training and certification*”.

“*Our premises do not offer privacy, you can’t give an injection in the eyes of everyone, pharmacies would need a new design and there is also need for a quiet room equipped to handle emergencies, for example anaphylaxis and sedation*”.

“*I think we lack confidence in doing our duties. We are too scared to bend the law and we always refer. I personally fail to make basic simple decisions that help the patient and simply refer for the fear of responsibility*”.

“*There is need to work out reimbursement models for pharmacists to be remunerated for these services. They should not be for free because remember the pharmacist is using his professional knowledge and time and the fee charged should promote access to health and not hinder or cast-off patients*”.

#### 3.1.5. Theme 5: Current Scope of BPharm Degree

Pharmacists indicated that the BPharm (Hons) degree curriculum is now out of touch with current pharmacy practice expectations and should be reviewed to make it patient centric.

“*The BPharm (Hons) 1 pharmacy degree was supposed to be reviewed after every 4 to 5 years. The last review was done in 1994 and that’s more than 20 years ago. The degree has definitely outlived its usefulness*”.

“*There is need for a patient focused curriculum to meet patient needs. More focus of the degree should be on clinical pharmacy and pharmacy practice experiences and not manufacturing*”.

“*I think we need to shift from viewing pharmacists as technical people designed to count tablets into professionals who are patient centred. The degree curriculum does not equip the Pharmacists with terminal competencies supporting an extended scope of practice*”.

Pharmacists also highlighted some sort of disconnect between pharmacists and policy makers on the future direction of pharmacy.

“*Pharmacists should be more clinical and hence more clinical roles should be added to the pharmacist duties in the undergraduate curricula. The policy makers want pharmacists to be more technical so that they can manufacture more medicines locally. They cannot support pharmacists giving vaccines when they believe that pharmacists can make medicines locally thereby reducing the import bill*”.

#### 3.1.6. Theme 6: Desired Pharmacist Role in UHC

Participants indicated that pharmacists need to do more to help patients’ access healthcare in the local communities they work from. Pharmacists highlighted that they should be able to change drug dosage/formulation, renew or extend a prescription, order and review laboratory tests, prescribe in minor ailments and common diseases such as HIV/AIDS, and administer vaccines and immunisations. It was also suggested that Pharmacies in the rural areas should be working as community health centres, where patients can get information about health, immunisations and vaccines.

“*I will be very happy to see pharmacists adopt all the above extended services that we have been discussing. The pharmacist has to leave dispensing of medications to pharmacy technicians and dispensary assistants*”.

“*The pharmacist needs to be involved in direct patient care such as identifying and managing drug therapy problems, communicating with the physician, prescription extension, ordering and reviewing laboratory data and administration of vaccines and immunisations*”.

“*The pharmacy should be a community health centre where people can access health with easy. The pharmacist needs to be more clinical and patient oriented to achieve universal health coverage*”.

### 3.2. Document Review of the BPharm Curriculum in Each School

BPharm (Hons) 1 programme has 2545 total learning hours whilst BPharm (Hons) 2 has 2580 h. Table 2 shows that BPharm (Hons) 1 has 542 h (21%) on social/behavioural/administrative pharmacy sciences and 441hrs (17%) on clinical sciences alone, while BPharm (Hons) 2 covers 270 h (10%) in clinical sciences alone and 240 h (9%) on social/behavioural/administrative pharmacy sciences. Both pharmacy programmes spent at least 46% on pharmaceutical sciences alone. BPharm (Hons) 1 curriculum has less basic and biomedical sciences (12%) compared to BPharm (Hons) 2 with 33.7%, hence BPharm (Hons) 2 curriculum is more industrial pharmacy focused whilst BPharm (Hons) 1 provides a broad programme of education in all pharmacy areas.

Extended services taught in BPharm (Hons) undergraduate degrees were depicted in Table 3. It was noted that administration of vaccines and prescribing in HIV/AIDS were extensively covered in BPharm (Hons) 1. Other services are not well defined and covered in both curricula.

## 4. Discussion

According to the participants of this study, the extension of pharmacist services is long overdue in Zimbabwe. During semi-structured interviews, pharmacists commented about the current traditional practice of pharmacy and demonstrated a strong willingness to offer extended pharmacist services in an effort to achieve universal health coverage. The pharmacists commented on the gaps in the undergraduate training programmes that did not provide enough clinical competencies and pharmacist confidence in discharging extended pharmacist services, the layout of community pharmacies that did not offer privacy, and the uncertainty in reimbursement models for pharmacists when they offer extended pharmacist services.

The review of the two pharmacy degree programmes offered in Zimbabwe showed totally different areas of emphasis as indicated in the credit hour distribution, but the graduates obtain the same qualification. The BPharm (Hons) 2 degree programme is industry focused with 80.2% of the time spent on pharmaceutical sciences and basic/biomedical sciences. BPharm (Hons) 1 has 38.6% of time spent on social/behavioural/administrative and clinical sciences compared to BPharm (Hons) 2 with only 19.8%. This shows that the BPharm (Hons) 1 pharmacy degree is more inclined towards public health programmes and patient care, while BPharm (Hons) 2 pharmacy degree is inclined to industry and less on patient care and public health. The review also showed that both pharmacy programmes spent almost 50% of total time on pharmaceutical sciences alone, thereby implying insufficient exposure of pharmacists in clinical sciences during their undergraduate studies to extend pharmacist services. Pharmacists are required to have the knowledge and skills extending beyond typical roles in the community pharmacy such as dispensing for them to be able to extend their services in primary healthcare. If the curriculum has gaps, these can be filled by recurriculization or through the provision of short courses or continuing professional development post-qualification to certify pharmacists to be proficient within clinical roles within a primary healthcare team.

Overall, the results suggested pharmacists’ positive overview on pharmacists’ involvement in direct patient management despite shortfalls in undergraduate training and exit level competencies required to extend pharmacist services. In the current study, pharmacists are keen to move from dispensing ready-made products to a more prominent role in the responsible provision of medicine therapy for the purpose of achieving definite outcomes that improve a patients’ quality of life. This process however is untenable currently due to the inadequacy of the current BPharm (Hons) curricula, which is not patient-centric and therefore does not equip graduates with terminal competencies supporting an extended scope of practice. The findings of this study are in line with the scope of pharmacy practice by the World Health Organisation, which now includes patient-centred care with all the cognitive functions of counselling, providing medicine information and monitoring medicine therapy as well as the technical aspects of pharmaceutical services including supply chain management [21]. By taking direct responsibility for individual patients’ medicine-related needs, pharmacists can make a unique contribution to the outcome of medicine therapy and to their patients’ quality of life. The International Pharmaceutical Federation in the year 2000 adopted the concept of the 7-star pharmacist from WHO in its policy on Good Pharmacy Education Practice (1997), which should be considered essential, minimum common expectations of pharmacists by healthcare systems world-wide [22,23]. The identified roles and responsibilities were pharmacist as a care giver, communicator, decision maker, teacher, lifelong learner, leader, and manager [22,23]. The pharmacist must therefore provide a link between the physician and patient, and hence should have complete knowledge about all the pharmaceuticals with recent updates and be confident while communicating with the physician and patient.

In an effort to extend pharmacist services, the South African Pharmacy Council in 2011 introduced a post-graduate diploma in Pharmacy for Authorised Pharmacist prescriber to teach pharmacists to diagnose and treat their patients using the medications listed in the primary healthcare essential medicine list [24]. In the United Kingdom, pharmacists were given the right to prescribe some exclusive medications and collaborate with doctors to develop a patient-specific clinical management plan [5,25]. However, the pharmacists have to attend an accredited supplementary prescribing course supervised by the Royal Pharmaceutical Society to be awarded a practice certificate [25]. The United States of America has transitioned from BPharm to PharmD as a first professional degree required for licensure to practice as a pharmacist. The PharmD curriculum was extended by 1 year that included additional pharmacotherapy and patient care coursework, plus extended experiential learning with specified activities emphasising clinical skills [18,20]. American pharmacists are taking on extended roles and are increasingly being recognised as the medication management experts of the healthcare team. In some states, pharmacists can independently or collaboratively prescribe any schedule 1 medicine, adapt and or manage prescriptions, order and interpret laboratory tests, and administer any medicine or vaccine by subcutaneous or intramuscular injection [18,20,26].

There are a few barriers that inhibit these extended services. For example, it is noted that the current pharmacist training is out of date with patient needs and confidence issues with some community pharmacists about the potential of these extended services to improve patients’ quality of life. The results of this study show that the Zimbabwe pharmacy curricula, namely BPharm as the only first professional degree and a prerequisite for licensing to practice the profession of pharmacy in Zimbabwe, has outlived its usefulness in terms of extending pharmacist services in patient care. In this study, the BPharm degree was not designed to absorb extended pharmacist services and pharmaceutical care but very useful in non-direct patient care areas such as pharmaceutical industry and marketing. In order to extend pharmacist services in primary healthcare, there is need for community pharmacists to enhance their clinical skills in diagnostic and therapeutic knowledge. A clinical-based era has emerged in pharmacy practice globally [27], as responding to fulfil the demand of the society. Therefore, pharmacists must enhance their therapeutic knowledge and skills to perform quality extended services in community pharmacy settings.

The WHO states that to make universal health coverage a reality, we need: individuals and communities who have access to high quality health services so that they can take care of their own health and the health of their families; skilled health workers providing quality, people-centred care; and policy makers committed to investing in universal health coverage [7]. In this study it is clear that pharmacists appreciate the need to extend roles of the pharmacist to patient care focus, however, pharmacists already in practice are mainly educated on the basis of the old paradigm of pharmaceutical product focus as demonstrated by the results of the curricula review. If these pharmacists are to contribute effectively to the new patient-centred pharmaceutical practice, they must have the opportunity to acquire the new knowledge and skills for their new role. There is an increasing global trend for example in countries towards transitioning from BPharm to the M. Pharm or PharmD degree as their entry-level qualification to practice as a pharmacist [28,29]. In Egypt, the PharmD is the minimum requirement to be licenced as a pharmacist [28,29]. In Ghana, a year top-up programme was rolled out in the 2017/18 academic year for practising pharmacists with the BPharm qualification to obtain the PharmD qualification, which will soon become the minimum degree for licensure [29]. Our study findings show that pharmacists are eager to extend their services to pharmaceutical care but lack the opportunity to acquire the new knowledge and skills for the extended pharmacist role. The traditional Master of Science in Pharmacy (Msc Pharm) and PhD programmes offered in Zimbabwe have no experiential or clinical practice and hence prepare students for teaching and research careers and not pharmaceutical care in the community.

The movement towards patient-care approach encompasses the opportunity for pharmacists to change and improve patient outcomes as integral active members of the patient care team. This study demonstrates that Zimbabwe pharmacy curricula has not been sufficiently updated to reflect new roles, which have helped perpetuate the undervalued status of pharmacists in the healthcare team. The pharmacy curricula emphasise more on the technical aspects of pharmacy rather than on professional practice. The major economic and political forces affecting the Zimbabwean healthcare system also have an impact on the practice of pharmacy [30]. As a result, radical changes are needed in pharmaceutical education. The role and function of pharmacists and pharmaceutical staff need to be reappraised and the education outcomes of the evolving pharmacy curriculum should be clearly defined. The educational change will not only require extensive curriculum revision and restructuring but also a major commitment to faculty development to prepare teachers to educate pharmacists in a different way. Education outcomes should include pharmaceutical care with the provision of patient-centred care, systems management of resources, medication use systems, and ensuring effective and quality public health.

### 4.1. Study Limitations

A limitation of this study is that other perspectives from, e.g., lecturers from the two pharmacy schools, officials from the Pharmacists Council of Zimbabwe, and policymakers were not captured. These stakeholders may have differing views or additional views on the expanded role of pharmacists under UHC. By including such stakeholders there would be more buy-in and less perception of threat to extending the role of pharmacists.

### 4.2. Recommendations

This study will be useful to make new policies and may bring transformation in pharmacy education at all levels. To develop patient care aspects, curriculum development teams and statutory authorities in Zimbabwe should start thinking of including these aspects in curricula at all levels. As part of a solution, it would be useful to have an alternative curriculum line focusing on patient care. An example might be an M. Pharm (pharmacy practice) or PharmD. This would give a positive scope for the development of the profession.

The Pharmacists Council of Zimbabwe needs to prescribe regulations governing the pharmacy curriculum leading to a registered pharmacist so that uniform standards are maintained throughout the country. Zimbabwe should not continue with all the different types of pharmacy programmes with wide variation leading to a registered person with the same title Pharmacist. This creates huge knowledge gaps within the profession in those individuals with the same professional titles and also increase the chances for inter-professional disputes [31].

There is also a need to conduct studies on patients and other healthcare professionals such as doctors and nurses to gather their perspectives on pharmacists, extending their roles to meet the needs of universal health coverage. Since pharmacists’ practices are supposed to be continuous with those of other healthcare professionals, their input and support will guarantee universal health coverage.

## 5. Conclusions

Zimbabwean pharmacists are in an excellent position to meet the needs of UHC by assuring the safe and effective use of medicines. To do so, pharmacists must assume greater responsibility than they currently do for the management of medicine therapies for the patients they serve. While supervision of the routine medicine distribution process must remain the responsibility of pharmacists, their direct involvement in medicine distribution must decrease since these routine activities can be handled by qualified pharmacy assistants. Thus, pharmacists’ responsibilities must be extended to include monitoring therapeutic progress and consulting with prescribers on behalf of patients to achieve UHC. The results show that pharmacists are very keen on extending their roles, but the training is not enough for them to feel competent.

There are serious gaps in the practice model that must be addressed before pharmacists will be in a position to properly deliver extended pharmacy services to Zimbabweans. Filling this gap must be top advocacy priority for pharmacy associations such as the Pharmaceutical Society of Zimbabwe and the Retail Pharmacists Association. Some of the barriers to extended pharmacist services include:

1. Lack of standardization or uniformity in the undergraduate degree programmes leading to be a registered pharmacist
2. Lack of confidence by some front-line pharmacists
3. Lack of adequate compensation to perform the extended service
4. Lack of collaboration or a team-based approach with other healthcare providers.

This study is the first of its kind in Zimbabwe, providing evidence to professional associations, statutory bodies, and policy makers for making plans to utilise the pharmacist workforce in an effort to achieve universal health coverage.

## Figures and Tables

**Table 1:** Demographic characteristics of the study participants (*n* = 10).

	Characteristics	Frequency
Age (years)		
	31–40	5
	41–50	3
	>51	2
Gender		
	Male	8
	Female	2
Qualifications		
	BPharm	8
	PharmD	1
	MPhil	1
Experience (Years)		
	10–14.	3
	15–20	3
	>21	4
Position in pharmacy		
	Employee manager	7
	Employee owner	3
Geographical location of pharmacies		
	Urban area	8
	Rural area	2

**Table 2:** Comparison of total time spent in each core content area, in number of hours in each study programme for BPharm (Hons) 1 and BPharm (Hons) 2.

Core Area	BPharm (Hons) 1		BPharm (Hons) 2	
	Hours	%	Hours	%
Basic and Biomedical Sciences	305	12	870	33.7
Pharmaceutical Sciences	1257	49.4	1200	46.5
Social/Behavioural/Administrative Sciences	542	21.3	240	9.3
Clinical Sciences	441	17.3	270	10.5
Total Hours	2545	100	2580	100

**Table 3:** Course content that teach students on Extended Pharmacist Services.

Extended Pharmacist	BPharm	BPharm	Comments
Service	(Hons) 1	(Hons) 2	
Administration of vaccines and immunisations	Yes	No	BPharm(Hons) 1 is thorough on the theoretical aspects of immunological products and vaccines but does not include practical aspects of their administration
Prescribing in HIV/AIDS	Yes	No	BPharm(Hons) 1 is exhaustive on the theoretical management of patients with HIV/AIDs
Change drug dosage/formulation	No	No	This extended service is not well defined in both pharmacy curricula
Order and review laboratory data	No	No	This extended service is not well defined in both pharmacy curricula
Review or extend a prescription	No	No	This service is not well defined in both pharmacy curricula
Prescribe in minor ailments	No	No	This service is not well defined in both pharmacy curricua
Smoking cessation programs	No	No	This extended service is not available in both programmes
Drug and substance abuse	No	No	This extended service is not available in both programmes

## Data Availability

All data upon which conclusions were drawn are within the manuscript in the form of tables and verbatim quotes.
